# Subclinical cardiac abnormalities in children with biliary atresia correlate with outcomes after liver transplantation

**DOI:** 10.3389/fped.2023.1174357

**Published:** 2023-03-30

**Authors:** Tingting Li, Xinzhe Wei, Xiaoye Hao, Xuying Ye, Chao Li, Qi Li, Zhuqing Li, Wei Gao, Chengzhi Lu

**Affiliations:** ^1^ The First Central Clinical School, Tianjin Medical University, Tianjin, China; ^2^ Pediatric Transplant Department, Tianjin First Central Hospital, Tianjin, China; ^3^ The Key Subject of Tianjin First Central Hospital, Tianjin, China; ^4^ Ultrasound Department, Tianjin First Center Hospital, Tianjin, China; ^5^ Department of Cardiology, Tianjin First Center Hospital, Tianjin, China; ^6^ School of Medicine, Nankai University, Tianjin, China

**Keywords:** subclinical cardiac abnormalities, children, biliary atresia, liver transplantation, outcomes

## Abstract

**Objective:**

There are subclinical cardiac abnormalities (SCA) in children with biliary atresia (BA). However, data on the consequences of these cardiac changes after liver transplantation (LT) remain controversial in the pediatric field. We aimed to determine the relationship between outcomes and the subclinical cardiac abnormalities in pediatric patients with BA based on two-dimensional echocardiography (2DE) parameters.

**Methods:**

A total of 205 children with BA were enrolled in this study. The relationship between 2DE parameters and outcomes, including death and serious adverse events (SAE) after LT, was analyzed by regression analysis. Using receiver operator characteristic (ROC) curves to determine the optimal cut-off values of 2DE parameters for outcomes. Differences in the AUCs were compared using DeLong's test. The Kaplan -Meier method with log-rank testing was used to evaluate survival outcomes between groups.

**Results:**

Left ventricular mass index (LVMI) and relative wall thickness (RWT) were found to be independently associated with SAE (OR: 1.112, 95% CI: 1.061 − 1.165, *P* < 0.001 and OR: 1.193, 95% CI: 1.078 − 1.320, P = 0.001, respectively). The cutoff value of LVMI for predicting the SAE was 68 g/m2.7 (AUC = 0.833, 95% CI 0.727-0.940, P < 0.001), and the cutoff value of RWT for predicting the SAE was 0.41 (AUC = 0.732, 95% CI 0.641-0.823, P < 0.001). The presence of subclinical cardiac abnormalities (LVMI > 68 g/m2.7, and/or RWT > 0.41) was associated with lower patient survival (1-year, 90.5% vs 100.0%; 3-year, 89.7% vs 100.0, log-rank P = 0.001). and higher incidence of SAE events.

**Conclusions:**

Subclinical cardiac abnormalities were correlated with post-LT mortality and morbidity in children with BA. LVMI can predict the occurrence of death and serious adverse events after liver transplantation.

## Introduction

Biliary atresia (BA) is an obstructive biliary disease, which is the main cause of neonatal cholestasis. It develops quickly to secondary biliary cirrhosis and results in mortality within the first two years of life if not treated (1). Currently, in most pediatric centers BA is the most common indication for liver transplantation (LT) (2).

It is believed that children with end-stage liver disease display cardiac structural changes associated with cirrhotic cardiomyopathy (CCM) (3). CCM is often a subclinical cardiac dysfunction in patients with liver cirrhosis, characterized by blunted contractile stress response, attenuated diastolic relaxation, and electrophysiological abnormalities without known cardiovascular disease (4). At the 2005 World Gastroenterology Congress, a group of experts proposed the diagnostic criteria for adult CCM based on the Doppler echocardiogram for the first time, including lower ejection fraction (<55%), an E/A ratio < 1, isovolumetric relaxation time > 80 ms, E-wave deceleration time > 200 ms, a left atrial volume increase, and increased myocardial mass (5). The existence of CCM leads to an increase in mortality and morbidity after liver transplantation (6).

Unlike in adults, the diagnostic criteria for pediatric CCM have not been established or validated, CCM in children is still relatively unrecognized and understudied (7). At present, there are few studies in the pediatric field that associated post-LT outcomes with cardiac changes associated with CCM. Among the first studies in children, Desai et al. found that up to 70% of patients with BA awaiting LT had identifiable functional and/or structural abnormalities detected on two-dimensional echocardiography (2DE) (8). In their follow-up study published by Gorgis et al. eight years later, they found that the specific 2DE parameters of pediatric CCM were correlated with peri-transplant outcomes, with a high incidence rate of morbidity and mortality (9). This finding is in contrast to the study by Junge et al. who documented a prolonged hospital stay but no increased morbidity and mortality in children with CCM (10). So, further evaluation of the influence of CCM on LT in children is required.

At present, the definition for pediatric CCM is proposed using 2DE parameters, principally including left ventricular mass index (LVMI) and relative wall thickness (RWT). In previous medical literature, no study has been reported about cardiac change associated with cirrhosis and its post-LT outcomes in Chinese pediatric patients. Therefore, we conducted this study to identify the influence of subclinical cardiac abnormalities (SCA) on LT and ascertain the prognostic value of LVMI and RWT for the outcomes after LT in children with BA at a large pediatric LT center.

## Materials and methods

### Study population and design

**Figure 1:**
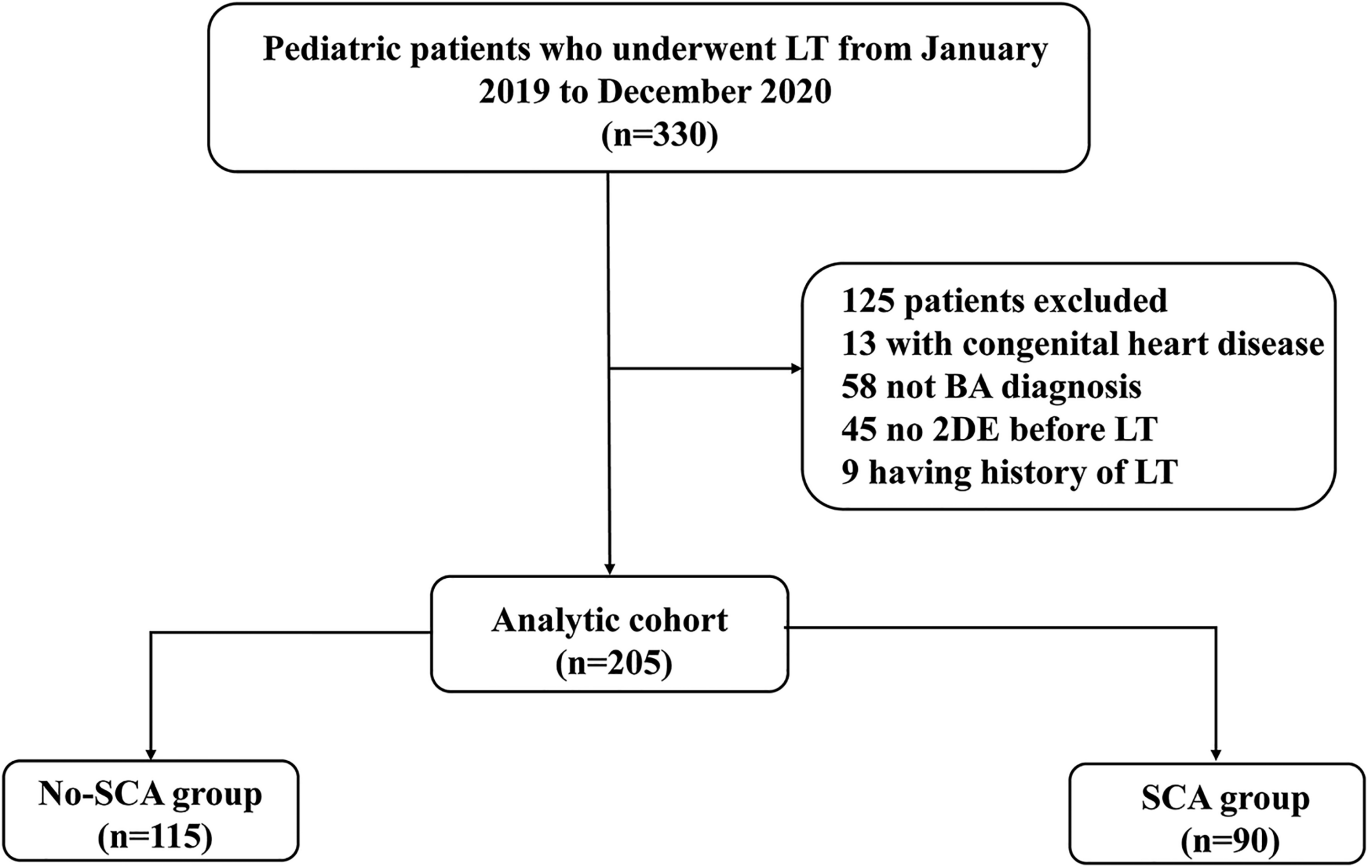
Study flow chart.

Clinical and demographic characteristics, as well as the outcomes, were extracted from a prospectively maintained database. In this retrospective cohort study, we screened 272 consecutive pediatric patients with BA who underwent LT from January 2019 to December 2020 at the Tianjin First Central Hospital, China. The exclusion criteria included the following: (i) Patients with congenital heart disease, including patent ductus arteriosus, ventricular septal defect, atrial septal defect and pulmonary stenosis;(ii) Having history of LT; (iii) lack of the data of 2DE before LT. A total of 205 patients were enrolled in this study. The study plan of enrollees is shown in Figure 1. The study adhered to the Helsinki Declaration and was approved by the institutional Ethics Committee of Tianjin First Central Hospital. (2021N168KY).

### Patient data

Data regarding age at LT, sex, history of a Kasai portoenterostomy (KPE), Pediatric End-Stage Liver Disease score (PELD) at time of LT, laboratory data including serum alanine aminotransferase (ALT), aspartate aminotransferase (AST), gamma-glutamyl transpeptidase (GGT), albumin, total bilirubin levels (TB), international normalized ratio (INR), prothrombin time (PT), platelet count (PLT), and C-reactive protein (CRP) were recorded. Body mass index (BMI) was indexed as the weight divided by the square of the height (kg/m^2^).

### Echocardiographic

To evaluate the structural and functional state of the heart, a complete transthoracic echocardiography including 2D, M-Mode, and Doppler echocardiography was performed in all patients using Philips iE33 ultrasound equipment. The key parameters that determined the LV geometry in the 2DE report were measured: LV end-diastolic diameter (LVIDd), LV posterior wall thickness during diastole (LVPWTd), and interventricular septum thickness during diastole (IVSd). The LV mass (LVM) was calculated based on the Devereux formula according to the American Society of Echocardiography guidelines (11). LVMI was indexed using height^2.7^ (g/m^2.7^). RWT was calculated as follows: (IVSd+LVPWd)/LVIDd. Furthermore, the E/A ratio (a ratio of early and late ventricular filling) was evaluated.

### Follow up

Outcomes were measured using patient survival rate and the occurrence of any component of the SAE (9) after LT. The SAE comprised the following: (i) loss of graft due to re-transplantation; (ii) hepatic artery and portal vein thrombosis; (iii) organ support treatment is required, including mechanical ventilation due to respiratory failure, vasoactive drug support or continuous renal replacement therapy (CRRT); (iv) Spontaneous hemorrhage (including gastrointestinal or intracranial hemorrhage) causes hemorrhagic shock. These parameters were chosen because they may seriously affect the patient's survival and postoperative outcomes after LT.

All patients were followed up from the time of transplant to death or the date of November 11, 2021.The median follow-up time after LT was 24 months (range, 0–34 months).

### Statistical analysis

The normal distributed continuous variables are shown as  ± S and were analyzed using Student's *t*-test. The non-normally distributed continuous variables are expressed as median (quartile 25, quartile 75) and were analyzed using the Mann–Whitney *U* test. The categorical variables are presented as number (n) and proportion (%) and were compared using Chi-square or Fisher's exact test. Univariate analysis was used to compare the baseline characteristics of patients with and without SAE, Statistically significant variables (*P *< 0.05) were included in multivariate logistic regression analysis to analyze the independent association between variables and SAE. The laboratory values such as bilirubin, INR, and albumin, which could reflect liver function, were included in the PELD score, so we did not include other laboratory values. The optimal cut-off values for each parameter and the diagnostic criteria's clinical efficacy were evaluated using receiver operator characteristic (ROC) curves. Differences in the AUCs were compared using DeLong's test. Survival outcomes between groups were compared using the Kaplan-Meier method and log-rank test. Data analysis was performed using SPSS (version 26.0, IBM Corp, Armonk, NY, USA) and MedCalc (version 19.1, MedCalc Software bvba, Ostend, Belgium; https://www.medcalc.org; 2019). A two-sided *P* value < 0.05 was considered to be statistically significant.

## Results

### General characteristics of the patients

A total of 205 pediatric BA patients with a median age level of 7.80 months at transplant participated in the study. 153 (75.0%) patients had a history of KPE. The median level of the PELD score was 16. Among the 205 patients, 25 (12.2%) had suffered SAE after LT, and 180 (87.8%) did not. Patients in the SAE group were younger at the time of LT than those in the No-SAE group (*P *= 0.045). Compared with the No-SAE group, the SAE group had higher LVMI, RWT, and PELD score (*P* < 0.001). The incidence of death was more common in the SAE group than in the No-SAE group (32.0% vs. 0.6%, *P *< 0.001). While EF and FS for systolic function and E/A ratio for diastolic function did not differ significantly between the two groups (*P* > 0.05). Other parameters showed no statistical difference between the two groups (*P > *0.05). As shown in Table 1.

**Table 1:** Baseline characteristics of SAE and No-SAE patients.

	All (*n* = 205)	No SAE (*n* = 180)	SAE (*n* = 25)	*P*
Age (months)	7.80 (6.10–12.73)	8.08 (6.09–14.90)	7.07 (6.13–8.17)	0.045
Sex, *n* (%)				0.376
Male	106 (51.7)	91 (50.6)	15 (60.0)	
Female	99 (48.3)	89 (49.4)	10 (40.0)	
BMI (Kg/m^2^)	16.2 ± 1.7	16.2 ± 1.7	16.2 ± 2.0	0.972
KPE, *n* (%)	153 (74.6)	135 (75.0)	18 (72.0)	0.747
PELD	16 (8–23)	16 (7–22)	22 (14–29)	0.002
LVM (g)	19.0 (15.3–24.8)	19.0 (15.3–24.6)	21.8 (17.7–24.8)	0.166
LVMI (g/m^2.7^)	57 (48–68)	56 (47–65)	74 (69–80)	<0.001
RWT	0.38 (0.34–0.42)	0.38 (0.33–0.42)	0.42 (0.40–0.48)	<0.001
FS (%)	33 (32–35)	33 (32–35)	33 (32–34)	0.535
EF (%)	64 (62–66)	64 (62–66)	64 (62–66)	0.874
E/A	1.31 (1.16–1.50)	1.31 (1.17–1.51)	1.19 (0.88–1.41)	0.096
Death, *n* (%)	9 (4.4)	1 (0.6)	8 (32.0)	<0.001

KPE, Kasai portoenterostomy; PELD, pediatric end-stage liver disease score; LVMI, left ventricular mass index; RWT, relative wall thickness; EF, left ventricular ejection fraction; FS, fraction shortening; SAE, serious adverse events.

### Multivariate logistic regression analysis for SAE and death

After adjusting for potential confounding factors using a multivariate logistic regression model, LVMI and RWT were found to be independently associated with SAE (odds ratio [OR]: 1.112, 95% confidential interval [CI]: 1.061–1.165, *P < *0.001 and OR: 1.193, 95% CI: 1.078–1.320, *P *= 0.001, respectively). Only LVMI was independently associated with death (OR: 1.045, 95% CI: 1.013–1.078, *P *= 0.005). As shown in Table 2.

**Table 2:** Multivariate logistic regression analysis for SAE and death.

	SAE		Death	
	OR (95% CI)	*P*	OR (95% CI)	*P*
Age (months)	0.991 (0.892–1.101)	0.866	0.952 (0.848–1.068)	0.402
LVMI (g/m^2.7^)	1.112 (1.061–1.165)	<0.001	1.045 (1.013–1.078)	0.005
RWT	1.193 (1.078–1.320)	0.001	1.050 (0.954–1.156)	0.316
PELD	1.081 (1.020–1.146)	0.008	1.033 (0.970–1.100)	0.312

LVMI, left ventricular mass index; RWT relative wall thickness; PELD, *p*ediatric end-stage liver disease score; SAE, serious adverse events.

### Predictive performance for SAE among the different parameters and models

**Figure 2:**
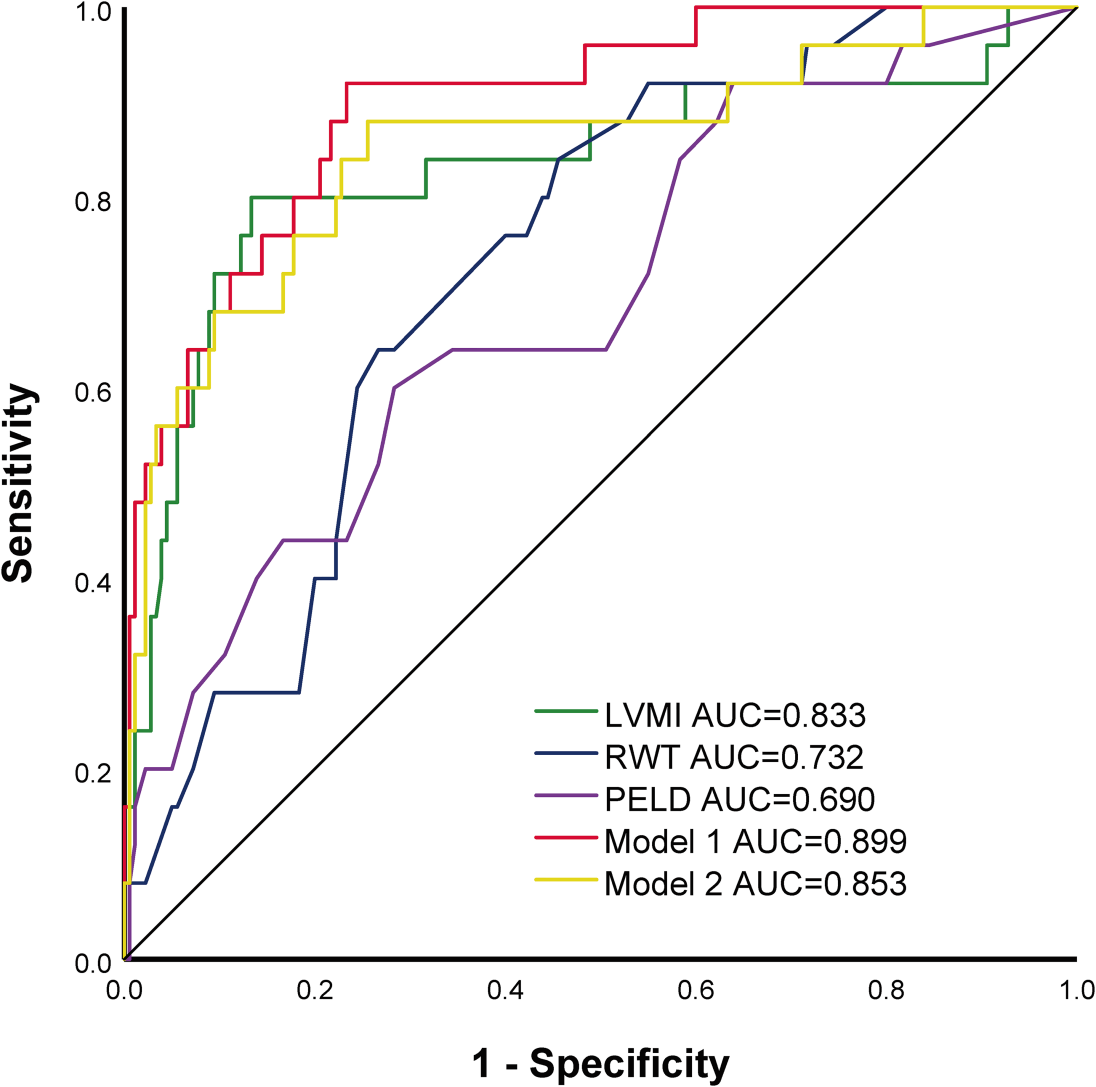
Receiver-operating characteristic (ROC) curve of different parameters and models for SAE.

The cutoff value of LVMI for predicting the SAE was 68 g/m^2.7^, with a sensitivity of 76.0%, specificity of 87.8% [area under the curve (AUC) = 0.833, 95% CI 0.727–0.940, *P *< 0.001], and the cutoff value of RWT for predicting the SAE was 0.41, with a sensitivity of 64.0%, specificity of 73.3% (AUC = 0.732, 95% CI 0.641–0.823, *P *< 0.001). Compared with PELD, LVMI showed improved AUCs in the prediction of SAE. (LVMI AUC: 0.833, 95% CI 0.727–0.940, *P* < 0.001 vs. PELD AUC 0.690, 95% CI 0.578–0.803, *P* = 0.002, respectively). Compared with LVMI, the model 1 constructed by LVMI, RWT, and PELD and the model 2 constructed by LVMI and PELD didn't significantly improve the prediction ability of SAE (Model 1 AUC: 0.899, 95% CI 0.836–0.962, *P* < 0.001 and Model 2 AUC: 0.853, 95% CI 0.760–0.946, *P* < 0.001). (As shown in Figure 2 and Table 3).

**Table 3:** Predictive performance among the different parameters and models.

	AUC	95%CI	Sensitivity (%)	Specificity (%)	*P*	*P1*	*P2*
LVMI	0.833	0.727–0.940	76.0	87.8	<0.001	0.087	
RWT	0.732	0.641–0.823	64.0	73.3	<0.001	0.580	
PELD	0.690	0.578–0.803	60.0	71.7	0.002		
Model1	0.899	0.836–0.962	92.0	76.7	<0.001	<0.001	0.059
Model2	0.853	0.760–0.946	88.0	74.4	<0.001	0.009	0.440

LVMI, left ventricular mass index; RWT relative wall thickness; PELD, *p*ediatric end-stage liver disease score; Model 1 constructed by LVMI, RWT, and PELD; Model 2 constructed by LVMI and PELD; P1, p-values between PELD and other parameters; P2, p-values between LVMI and other parameters.

### Predictive value of LVMI combined with RWT

**Figure 3:**
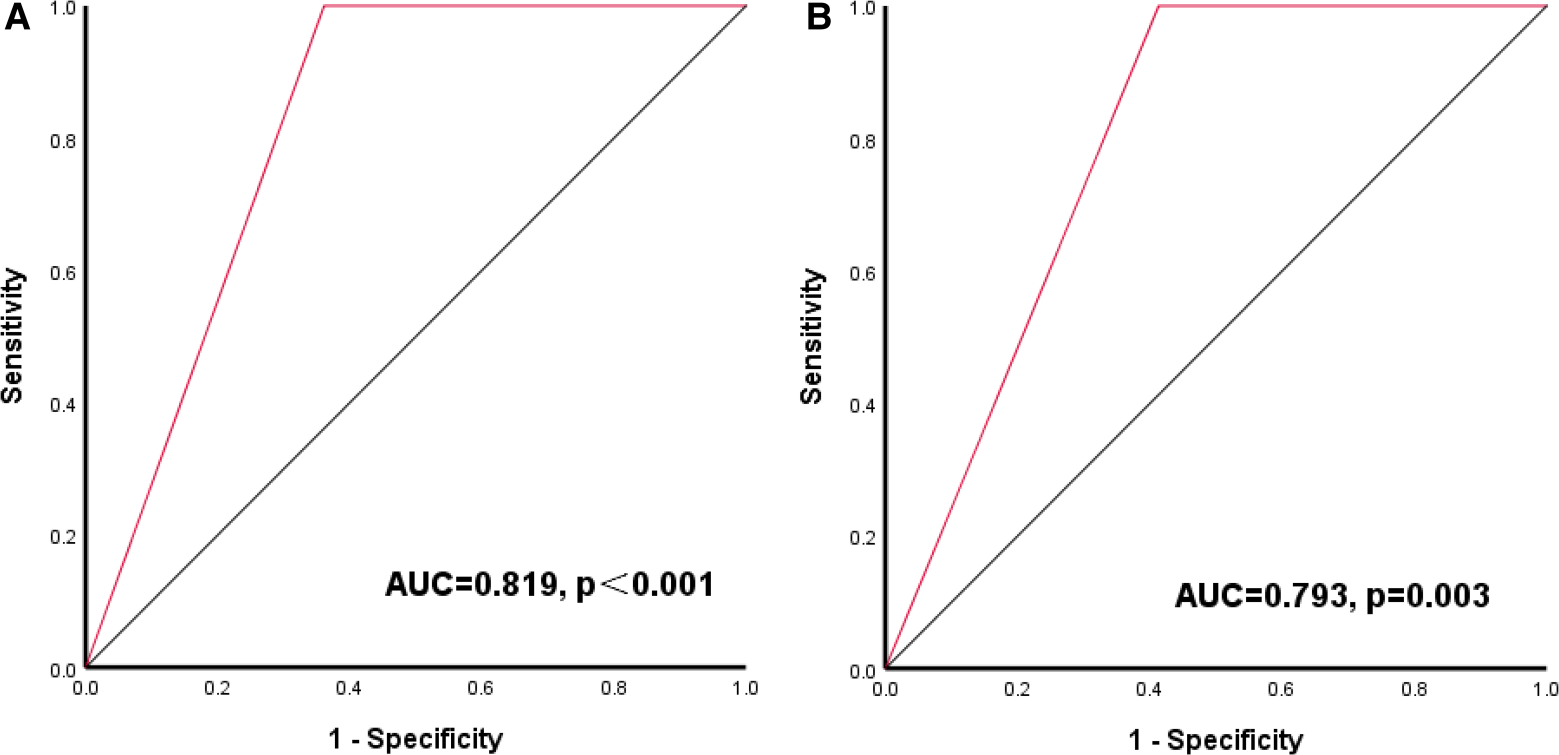
Receiver-operating characteristic (ROC) curve of LVMI combined with RWT for outcomes after LT (**A**) ROC curve of LVMI combined with RWT for SAE; (**B**) ROC curve of LVMI combined with RWT for death.

Our results showed that LVMI and RWT are predictive of the outcomes after LT in children with BA. These two parameters are an important part of the CCM diagnostic criteria. We used the appeal optimal cut-off value as the diagnostic threshold (LVMI > 68 g/m2.7, and/or RWT > 0.41) of subclinical cardiac abnormalities for the next study. The presence of subclinical cardiac abnormalities can predict the occurrence of SAE after LT, with a sensitivity of 100.0%, specificity of 63.9% [AUC = 0.819 (95% CI, 0.760–0.879), *P *< 0.001]. Similarly, the presence of subclinical cardiac abnormalities can also predict the occurrence of death after LT, with a sensitivity of 100.0%, specificity of 58.7% [AUC = 0.793 (95% CI, 0.704–0.883), *P *= 0.003]. As shown in Figure 3.

### Baseline characteristics and outcomes associated with subclinical cardiac abnormalities

According to the optimal cut-off values of LVMI and RWT, 90 of 205 (43.9%) patients had subclinical cardiac abnormalities (SCA). Patients in the SCA group were younger at the time of LT than those in the No- SCA group (*P *= 0.027) and had a higher PELD score (*P *= 0.015). E/A ratio for the diastolic function was significantly lower in the SCA group than in the No-SCA group (*P < *0.001).While EF and FS for systolic function did not differ significantly between the two groups (*P* > 0.05). There were no statistically significant differences in other baseline characteristics (*P *> 0.05). There were a total of 9 deaths, all of which occurred in the SCA group. The survival rate in SCA group was significantly lower than that of No SCA group (*P* < 0.001). Compared with the No-SCA group, patients in the SCA group were more likely to suffer SAE after LT (*P* < 0.001). 25 suffered SAE after LT, including 1 retransplant, 16 requiring organ support therapy, 7 hemorrhages, and 5 thromboses (hepatic artery or portal vein), all coming from the SCA group. Patients in the SCA group also had longer ICU and total hospital stays than those in the non-SCA group (*P* < 0.05). As shown in Table 4.

**Table 4:** Comparison of characteristics between patients with and without SCA.

	All (*n* = 205)	No SCA (*n* = 115)	SCA (*n* = 90)	*P*
Age (months)	7.80 (6.10–12.73)	8.27 (6.10–19.75)	7.32 (6.10–9.30)	0.027
Sex, *n* (%)				0.209
Male	106 (51.7)	55 (47.8)	51 (56.7)	
Female	99 (48.3)	60 (52.2)	39 (43.3)	
BMI (Kg/m^2^)	16.2 ± 1.7	16.1 ± 1.7	16.4 ± 1.8	0.268
KPE *n* (%)	153 (74.6)	84 (73.0)	69 (76.7)	0.554
LVMI (g/m^2.7^)	57 (48–68)	52 (45–60)	67 (57–74)	<0.001
RWT	0.38 (0.34–0.42)	0.35 (0.32–0.38)	0.43 (0.40–0.48)	<0.001
FS (%)	33 (32–35)	34 (32–35)	33 (31–35)	0.193
EF (%)	64 (62–66)	64 (62–66)	64 (62–66)	0.715
E/A	1.31 (1.16–1.50)	1.38 (1.20–1.56)	1.25 (1.12–1.36)	<0.001
PELD	16 (8–23)	15 (5–21)	19 (11–26)	0.015
ALT (U/L)	127.85 (71.10–209.10)	130.90 (68.05–217.70)	126.80 (80.40–198.00)	0.934
AST (U/L)	224.95 (134.30–372.55)	239.80 (126.95–381.60)	206.50 (147.40–364.50)	0.891
GGT (U/L)	268 (120–570)	229 (118–512)	303 (127–632)	0.115
Albumin (g/L)	34 ± 6	34 ± 6	34 ± 6	0.746
TB (umol/L)	226.65 (70.78–320.37)	220.50 (50.86–305.68)	228.73 (130.07–337.90)	0.367
INR	1.38 (1.13–1.77)	1.31 (1.12–1.69)	1.43 (1.14–1.81)	0.146
PT (s)	16.4 (13.2–24.2)	16.0 (13.3–26.3)	17.1 (13.1–22.6)	0.885
CRP (mg/L)	5.80 (1.97–15.27)	4.98 (1.51–13.92)	6.25 (2.79–18.42)	0.058
Platelet (×10^9^/L)	196 (130–291)	191 (120–282)	200 (142–315)	0.387
Death *n* (%)	9 (4.4)	0	9 (10.0)	<0.001
SAE *n* (%)	25 (12.2)	0	25 (27.8)	<0.001
Re-transplantation	1 (0.5)	0	1 (1.1)	0.439
Organ support	16 (7.8)	0	16 (17.8)	<0.001
Hemorrhage	7 (3.4)	0	7 (7.8)	0.003
Thrombosis	5 (2.4)	0	5 (5.6)	0.015
ICU stay (day)	2.0 (1.5–2.5)	2.0 (1.5–2.5)	2.0 (1.5–3.0)	0.046
Hospital stay (day)	25 (18–35)	22 (17–32)	29 (20–38)	0.004

SCA, subclinical cardiac abnormalities; BA, biliary atresia; KPE, Kasai portoenterostomy; LVMI, left ventricular mass index; RWT, relative wall thickness; EF, left ventricular ejection fraction; FS, fraction shortening; PELD, *p*ediatric end-stage liver disease score; ALT, alanine aminotransferase; AST, aspartate aminotransferase; GGT, gamma-glutamyl transpeptidase; TB, total bilirubin levels; INR, international normalized ratio; PT, prothrombin time; CRP, C-reactive protein; SAE, serious adverse events; ICU, intensive care unit.

**Figure 4:**
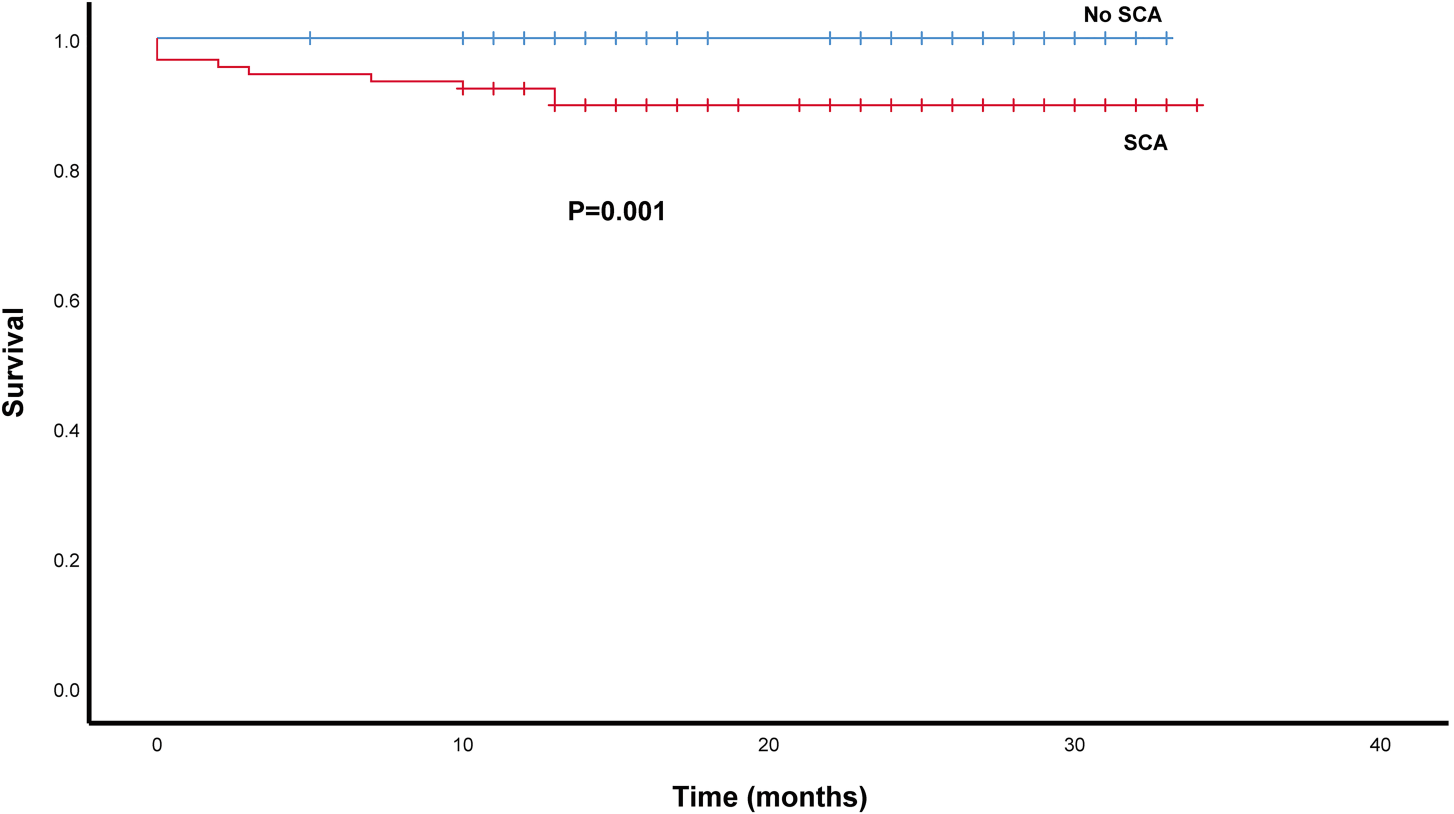
Survival analysis of SCA patients after LT using the kaplan–meier method. The patients survival rate was lower in the SCA group than in the non -SCA group (log-rank *P *= 0.001).

As depicted in Figure 4, we compared patient survival by the Kaplan–Meier method in the SCA and No SCA group. The SCA group had a lower patient survival rate than the No-SCA group (log-rank *P *= 0.001). The 1-year and 3-year patient survival of the SCA group vs. the No-SCA group were 90.5% vs. 100.0% and 89.7% vs. 100.0%, respectively. Notably, the survival difference between the No-CHD group and the CHD group mainly happened in the first 12 months after liver transplantation, and after that, the survival curves are parallel. Although the 3-year survival rate was lower in the SCA group vs. the No-SCA group, the cluster of deaths within the first 12 months is noteworthy, and further research is needed. We should concentrate on the 1-year survival rate of SCA group clinically.

## Discussion

As far as we know, this is the first study to describe subclinical cardiac abnormalities associated with pediatric BA from China. We found that specific 2DE parameters including LVMI and RWT can predict the poor outcomes after liver transplantation. Subclinical cardiac abnormalities were correlated with post-LT mortality and morbidity in children with BA. Patients in the SCA group had a lower survival rate and a higher incidence of SAE events, and it was also related to prolonged ICU and hospital stays. Notably, the survival difference between the SCA and No-SCA groups occurred primarily in the first 12 months after transplantation, so we should focus on the 1-year survival rate of SCA group clinically.

The median age at transplantation was 7.8 months in our study. It is known that in the mouse heart the development of cardiac alterations including hypertrophy and hyperdynamic contractility appears even within a few weeks of biliary tract injuries (12). And if so, the inclusion of very young patients with BA, a disease of biliary cirrhosis that occurs soon after birth made the results even more influential. This made it possible to infer that even patients with short-term cirrhosis may already have myocardial involvement, even subclinical.

In patients with cholestatic BA, cardiac remodeling can occur in three ways. First, there is hemodynamic stress and a chronic inflammatory state in pediatric patients with BA, which contribute to pathological cardiac hypertrophy, cardiomyocytes respond to a number of pathological stimuli by releasing chemokines, inflammatory cytokines, and damage-associated molecular patterns, further increasing the inflammatory response. This immune response can subsequently activate pro-hypertrophic and profibrotic pathways, resulting in heart hypertrophy and remodeling (13, 14). Second, increased circulating bile acids have been linked to cardiac dysfunction in preclinical models of cirrhosis, possibly by activating the bile acid receptors farnesoid X receptor and TGR5 (15). Third, other factors that may contribute to the cardiovascular abnormalities found in infants with cholestasis include nitric oxide, endogenous opioids, and endocannabinoids. These factors may work together to produce vasodilation and impair cardiovascular responses to sympathetic stimulation (16). The effects of cirrhosis on cardiac functions and two-dimensional echocardiography parameters have mostly been published in adult studies (17, 18). Although the number of studies about the cardiac effects of cirrhosis has increased recently, especially in children, the number of studies conducted in children is still lacking.

We have to emphasize the importance of identifying LVMI among children with BA. In our study, we found that LVMI can predict the occurrence of death and serious adverse events after liver transplantation. LVMI has a stronger predictive effect than RWT and PELD. And compared with LVMI, the model constructed by LVMI, RWT, and PELD and the model constructed by LVMI and PELD have no obvious advantages, this has not been reported in previous studies. There is no doubt that changes in cardiac structure and function remodeling have been observed in patients with biliary atresia. In the 2011 report of Desai et al., children with BA had significant increases in multiple 2DE parameters, notably left ventricle mass index and left ventricle wall thickness, similar to our findings (8). As far as we know, in the pediatric field only a few studies correlated post-LT consequences to cardiac changes associated with cirrhosis (9, 10). While, the existing data is controversial. Gorgis et al. have shown that the specific 2DE parameters of abnormal cardiac findings are related to peri-transplant outcomes, while Junge et al. did not find this. Our results are consistent with Gorgis. Therefore, this study is of prime value because it is one of the few studies in the literature indicating that the left ventricular mass index correlates with peri-transplant outcomes in children with BA. Further verification is needed in more prospective, multi-center studies in the future.

We also found that the combination of LVMI and RWT is predictive of the outcomes after LT in children with BA. Elevated LVMI and RWT are used to identify left ventricular hypertrophy (LVH), which has been related to poor outcomes, including death, in both adults and pediatric patients with cirrhosis, and has also been related to morbidity in other diseases (8, 18–20). At present, there are no established thresholds for determining LVH in children. In our study, we used the optimal cut-off values (LVMI > 68 g/m2.7, and/or RWT > 0.41) to identify the subclinical cardiac abnormalities (SCA). This is similar to the method used by Gorgis et al. to derive the definition of CCM for children (9). In our cohort, patients with subclinical cardiac abnormalities were at risk of worse post-LT outcomes than those without. Notably, the survival difference between the SCA group and the No-SCA group mainly occurred in the first 12 months after transplantation. Some researchers suggest that abnormal 2DE parameters associated with CCM may cause some problems in the early stage after LT, but the long-term prognosis is better (21, 22). This may be explained by the regression of subclinical cardiac change after LT. Studies have shown that in both adults and children, this cardiac abnormality can recover about one year after LT (9, 23).

It has been reported that changes in diastolic function first appeared in ESLD patients with cardiac abnormalities, while systolic function may be normal (24). Our results were consistent with that. In our study, EF and FS for systolic function did not differ significantly between the SCA and No SCA groups. While, E/A ratio for the diastolic function was significantly lower in the SCA group than in the No SCA group, even though these values didn't exceed the normal range. Systolic function is usually reserved for a very late course of disease, and this dysfunction could become evident clinically if pharmacological or physical stress tests were conducted. Compared with adults, pediatric patients with BA may have had greater cardiac reserves (25).

Before pediatric liver transplantation or at the time of transplantation, there are few parameters with solid evidence to predict outcome. The PELD score, as a recognized parameter for determining organ allocation and priority, has been recognized for its effectiveness in predicting the risk of death on the waiting list before transplantation (26). However, the PELD score has a limited ability to predict the postoperative mortality of pediatric patients (27). With the increase in organ allocation based on the PELD exception score, the fairness of the PELD score has been questioned (28). In our study, we found that elevated LVMI and RWT can predict poor outcomes after liver transplantation. And a recent study suggests that abnormal 2DE features are present early in life in patients with BA and usually occur at the age when BA is diagnosed (29). We surmise that if we compared these 2DE parameters to the whole pool of patients on a liver transplant waiting list, this extrahepatic feature could optimize organ allocation and direct clinical care in the future. Overall, our findings hope to draw attention to the presence of subclinical cardiac abnormalities in children with BA in clinical work. These findings complement the data of pediatric LT and will provide tools for future improvement.

## Limitations

We were aware of the limitations of our study. First, this was a single-center retrospective study, and the results may not be representative of patient populations in other centers. Second, we did not conduct regular monitoring of cardiac ultrasound after liver transplantation, so we could not understand the evolution of cardiac abnormalities in this patient population. Further verification needs more prospective, multi-center studies in the future.

## Conclusion

Subclinical cardiac abnormalities were correlated with post-LT mortality and morbidity in children with BA. LVMI can predict the occurrence of death and serious adverse events after liver transplantation.

## Data Availability

The raw data supporting the conclusions of this article will be made available by the authors, without undue reservation.
